# Cochrane Africa: a network of evidence-informed health-care decision making across sub-saharan Africa

**DOI:** 10.11604/pamj.2018.29.196.14521

**Published:** 2018-04-03

**Authors:** Lawrence Mbuagbaw, Pierre Ongolo Zogo, Tamara Kredo, Solange Durao, Taryn Young, Emmanuel Effa, Martin Meremikwu, Ameer Hohlfeld, Charles Wiysonge

**Affiliations:** 1 Centre for the Development of Best Practices in Health, Central Hospital, University of Yaoundé 1, Yaoundé, Cameroon; 2 Department of Health Research Methods, Evidence and Impact McMaster University, Hamilton, Canada; 3 Biostatistics Unit, Father Sean O’Sullivan Research Centre, St. Joseph’s Healthcare Hamilton, Hamilton, Ontario, Canada; 4 Cochrane South Africa, South African Medical Research Council, Cape Town, Western Cape, South Africa; 5 Division of Clinical Pharmacology, Faculty of Medicine and Health Sciences, Stellenbosch University, Cape Town, South Africa; 6 Centre for Evidence-based Health Care, Faculty of Medicine and Health Sciences, Stellenbosch University, Cape Town, South Africa; 7 Department of Medicine, University of Calabar, Calabar, Nigeria; 8 Cochrane Nigeria, Institute of Tropical Disease Research and Prevention, University of Calabar Teaching Hospital, Calabar, Nigeria; 9 Department of Paediatrics, University of Calabar, Calabar, Nigeria

**Keywords:** Cochrane Africa, evidence-informed healthcare, health outcomes, systematic reviews

## Abstract

Cochrane Africa is a network of researchers and health stakeholders who aim to support the use of high quality Cochrane evidence to improve health outcomes in Africa. It comprises a coordinating centre in South Africa, a Francophone hub directed from Cameroon, a Southern and Eastern Africa Hub directed from South Africa and a West Africa Hub directed from Nigeria. The network supports the engagement with healthcare decision makers to guide priorities, production of high quality context-relevant Cochrane systematic reviews, capacity building to conduct and use reviews, dissemination of evidence, knowledge translation, partnerships for evidence-informed healthcare and the creation of opportunities to expand the network.

## To the editors of the Pan African Medical Journal

On the 15^th^ September 2017, Cochrane Africa was launched at the Global Evidence Summit in Cape Town, South Africa [1]. This network is the result of coordinated efforts to build capacity for conducting systematic reviews and promote the use of best evidence to inform healthcare decision making on the African continent.

This network is a much needed response to the high burden of disease, health system challenges and limited research capacity in sub-Saharan Africa [2, 3]. Beginning in 2007, African collaborators came together to encourage the production of high quality systematic reviews that are relevant to Africa and to support their use to inform policy and practice. We have since then, informed many national and international guidelines, especially in malaria, tuberculosis and HIV.

Cochrane Africa (www.africa.cochrane.org) includes a coordinating centre at Cochrane South Africa, a Francophone hub directed from Cameroon, a West Africa Hub directed from Nigeria and a Southern/Eastern Africa Hub directed from South Africa.

The Network's goal of increasing the use of best evidence to inform healthcare decision making in sub-Saharan Africa is accomplished through five activities: producing context-relevant systematic reviews identified through a consultative process with end-users based on their needs, priorities and acknowledged research gaps; building capacity to conduct and use systematic reviews; advocating for the dissemination, translation and use of evidence; building partnerships to promote African-led evidence-informed healthcare and creating opportunities for the network to grow.

Historically, there has only been one Cochrane Centre in Africa, Cochrane South Africa, with a branch in Nigeria. Cochrane Africa represents a unique opportunity to apply known methods for supporting evidence uptake to address issues that plague African health services including limited research capacity, high disease burden and limited use of evidence in formulating and implementing policy. It offers a platform for much needed South-South collaboration, sharing of experiences and addressing some barriers to evidence use in Africa [4]. Our work includes efforts to connect with Francophone and Lusophone countries to build capacity and support the production of systematic reviews in languages other than English. Our strengths lie in the breadth of the network, many years of experience, individual and institutional capacity strengthening and stakeholder engagement.

## Conclusion

We invite interested parties (researchers, health care providers, journalists, policy-makers, consumers) to visit our website and join us to strengthen the network, add relevance to the work we do and reduce inequities in health research that lead to inequities in health outcomes [5].

## Competing interests

The authors declare no competing interest.
